# Protecting Our Children from Climate Change

**DOI:** 10.1289/ehp.1409165

**Published:** 2014-10-01

**Authors:** Linda S. Birnbaum, Kimberly Thigpen Tart

**Affiliations:** 1 Director, and; 2 Office of Policy, Planning, and Evaluation, National Institute of Environmental Health Sciences, National Institutes of Health, Department of Health and Human Services, Research Triangle Park, North Carolina, USA

This month, as we celebrate National Children’s Health Month with *Environmental Health Perspectives’* (*EHP*) annual issue focused on children’s health around the world, one particular nexus of concern has risen to the forefront: the current and potential impacts of climate change on children’s health. Although climate change has become a constant topic of scientific inquiry and political discussion, scant U.S. attention has been paid to what it may hold in store for some of the most vulnerable members of our society—the youngest. Recently, though, this has begun to change: The President’s Task Force on Environmental Health Risks and Safety Risks to Children, a federal interagency group that coordinates efforts on a variety of topics important to children’s health, and others such as the American Academy of Pediatrics have recognized the need to investigate and understand the risks that climate change poses to children so that we can take measures to protect children’s health. In July, the Subcommittee on Climate Change of the Task Force, co-led by the National Institute of Environmental Health Sciences (NIEHS), the U.S. Environmental Protection Agency (EPA), and the Department of Homeland Security, organized an Expert Consultation on the Effects of Climate Change on Children’s Health that engaged academic and government experts, children’s advocates, and leaders from federal agencies and the White House. NIEHS interest in and support for this topic represents a natural convergence of our institute’s investment in children’s health research with our efforts to examine the implications of climate change on the health of all people, but particularly those populations likely to be most vulnerable to its effects.

Children’s environmental health has been a priority of our institute for decades. Early work funded and conducted by the NIEHS formed the foundation of global understanding of the health effects on children of lead, mercury, arsenic, and other toxicants. More recent work has elucidated the mechanisms and effects of endocrine disruptors, pesticides, and allergens. In 1998, the National Toxicology Program (NTP) and the NIEHS established the Center for the Evaluation of Risks to Human Reproduction, known as CERHR. The center served until 2010 as a clearinghouse for scientific information on environmental agents that could affect human reproduction and development. In 2011, the center was subsumed into the current NTP Office of Health Assessment and Translation, which continues this important evaluation work more broadly, but with ongoing attention to children’s development and health. Also in 1998, the NIEHS and the U.S. EPA launched the Centers for Children’s Environmental Health and Disease Prevention Program. These co-funded centers comprise a national network of scientific and community leaders, health care providers, and government officials who share the goals of preventing and reducing childhood diseases and translating research findings to the affected communities and the broader public.

Our institute’s efforts to determine the potential health impacts of climate change are similarly long-standing. The NIEHS was named as the Department of Health and Human Services principal to the U.S. Global Change Research Program in the Global Change Research Act of 1990 (1990), and in that role now co-leads the program’s Interagency Crosscutting Group on Climate Change and Human Health. In 1990, NIEHS scientists participated in the first Intergovernmental Panel on Climate Change report (1990). In 1994, shortly after it became a monthly publication, *EHP* ran a three-part series on climate change and its potential impacts on communities (Alderson 1994; Breslin 1994; Manuel 1994) and has continued to report and publish important related research findings ever since. In 2009–2010, the NIEHS led, with the U.S. EPA, Centers for Disease Control and Prevention (CDC), and National Oceanic and Atmospheric Administration (NOAA), a federal interagency working group that published the landmark paper “A Human Health Perspective on Climate Change” (Interagency Working Group on Climate Change and Health 2010). Included in the 11 impact areas and associated research needs identified in the paper were many specific to children’s health and development. In 2011, the NIEHS launched a funding program to explore the impacts of climate change on vulnerable populations, including pregnant women and children.

Thus, it is not just a strong tradition of scientific efforts in such focused programs that the NIEHS brings to the table, but also a constantly growing knowledge base in a broad array of relevant fields including air pollution and allergies, toxic contamination, developmental origins of health and disease, health disparities, and postdisaster research and response, to name a few. Work in these areas is directly applicable to understanding and anticipating the effects of climate change on children. For example, knowledge of how the body’s mechanisms respond to air pollutants such as particulate matter and ozone can inform the potential for climate change to exacerbate asthma—a huge health burden among children, particularly urban minority and other disadvantaged children. An ever-expanding comprehension of the effects of pesticide exposures on children’s growth and development can be brought to bear on climate change issues such as predicting impacts of increased toxic exposures from extreme weather events, as well as changes in food production and security, particularly among indigenous cultures. New research that implicates exposure of pregnant mothers to floods as a potential factor in negative birth outcomes, the effects of which can extend into childhood and beyond, may benefit from the findings of research on the developmental origins of health and disease. And efforts to learn how best to quickly identify and respond to health impacts in the aftermath of disasters such as hurricanes, floods, and storms—all of which are expected to become more frequent and severe with climate change—may help to guide how we protect children from and improve their resilience to such events.

There has never been a greater need for investigations of climate change and dissemination of credible, understandable, and indisputable information on its potential to affect the health and lives of children. The NIEHS will continue to work with all of our partners, including those across the federal government, throughout our stakeholder community, and within our World Health Organization Collaborating Centre and global network, to foster such research and its translation to those who can use it to make sound public health decisions.

Climate change, like all other environmental health issues, is not a for/against proposition. The health of our planet and the health of the people who live on it are inextricably intertwined. Therefore, how we act today to understand and address climate change is intricately linked to the tomorrows our children, and our children’s children, will face. As the impact of climate change on our children’s futures and health increasingly becomes an issue of concern for the nation and the world, so will it continue to be an issue of concern for the NIEHS.
